# Exploring atypical timescales in the brain

**DOI:** 10.7554/eLife.45089

**Published:** 2019-02-05

**Authors:** Leonardo L Gollo

**Affiliations:** Systems Neuroscience GroupQIMR Berghofer Medical Research InstituteBrisbaneAustralia

**Keywords:** timescales, autism, brain activity, psychiatry, imbalances, ASD, Human

## Abstract

Identifying activity imbalances in specific brain regions may help to diagnose and treat psychiatric disorders.

**Related research article** Watanabe T, Rees G, Masuda N. 2019. Atypical intrinsic neural timescale in autism. *eLife* 8:e42256. doi: 10.7554/eLife.42256

The electrical activity of any region of the brain changes with time in a complex way that can be described as combinations of oscillations with different amplitudes, frequencies and phases. Different areas of the brain are also characterized by an intrinsic timescale that reflects the length of the time window over which the signals coming into that brain region are integrated (Mesulam, 1998; Honey et al., 2012).

Regions with short intrinsic timescales are usually located at the periphery of the brain network and are implicated in interactions between the brain and the external world, for example, perception and movement. Regions with long timescales are usually strongly connected hubs located at the core of the brain. They are important for regulating interactions between the brain and the body, such as emotions, mood and anxiety (Gollo et al., 2015). This gradient of timescales forms a hierarchy in brain dynamics that recapitulates the hierarchy in brain structure (Kiebel et al., 2008; Murray et al., 2014; Figure 1A).

**Figure 1.**
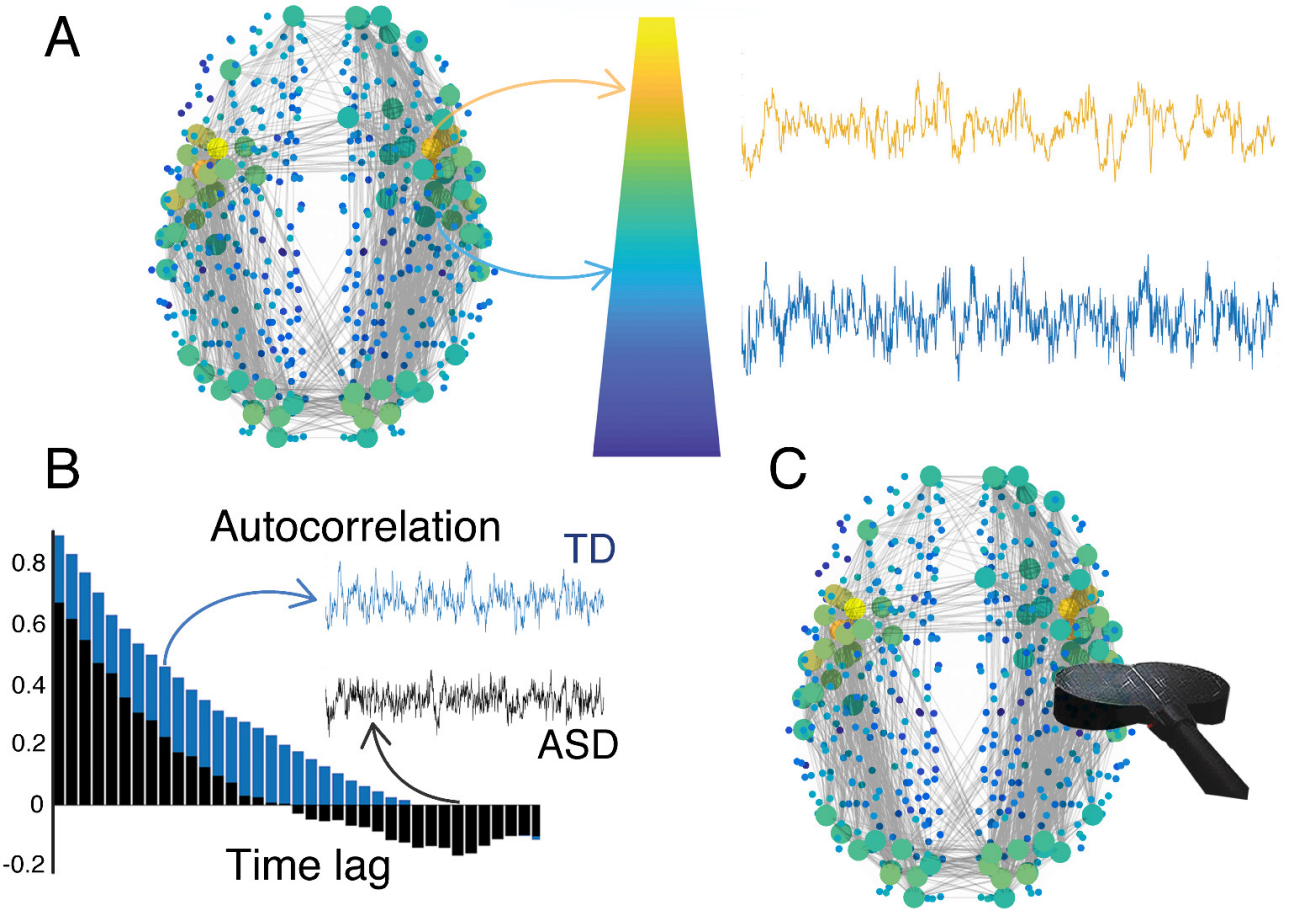
The hierarchy of timescales in the brain. (**A**) The brain integrates incoming information over different timescales that are characteristic for different regions. Such a hierarchy of timescales also mirrors a hierarchy in brain structure. Brain regions located at the top of the hierarchy are represented as large (yellow) circles and have longer timescales. They are located at the core and have strong connections to other brain regions. Brain regions located at the periphery are represented by small (blue) circles and have shorter timescales. (**B**) Watanabe et al. found that individuals with autism spectrum disorders (ASD, black) have different intrinsic timescales (quantified by the autocorrelation function) compared to typically developing individuals (TD, blue). These differences correlate with the severity of symptoms of ASD. (**C**) In the future, non-invasive brain stimulation (black coil) may be used to selectively modulate atypical brain regions to restore their intrinsic timescales. Brain figure adapted from Gollo et al. (2018).

This hierarchy of timescales also plays an important role in perception and many other behaviors, and modifications to these timescales can be detrimental to brain function (Kiebel et al., 2008; Murray et al., 2014; Heeger, 2017). Now, in eLife, Takamitsu Watanabe, Geraint Rees and Naoki Masuda report that changes in intrinsic timescales are associated with the symptoms of autism spectrum disorder in high-functioning individuals (Watanabe et al., 2019). Their study raises the question of whether the intrinsic timescales can be used as a biomarker for neuropsychiatric disorders and as a target for potential treatment therapies.

The researchers – who are based at the RIKEN Centre for Brain Science, University College London and the University of Bristol – used functional magnetic resonance imaging to measure intrinsic timescales in people with and without a high-functioning form of autism. The results revealed that people with this form of autism have atypically short timescales in primary sensory and visual areas, while a region called the caudate, which is implicated in sensorimotor coordination, showed a longer timescale (Grahn et al., 2008). This reinforces the theory that intrinsic timescales are central to brain function, and that imbalances in specific regions substantially affect the severity of symptoms in autism spectrum disorders (Figure 1B).

Intrinsic timescales can be estimated using simple autocorrelations, which may be used to identify biomarkers and to improve our understanding of diseases and treatment plans (Figure 1B). But further research is needed to fully comprehend the causes and implications of atypical intrinsic timescales. In people with autism, shorter timescales in regions of sensory and visual cortices could relate to a heightened sensory perception, which is consistent with an excessive expectation of changes in their environment (Lawson et al., 2017). Moreover, longer timescales in the caudate might also indicate a compensation strategy to cope with an overload of sensory input due to the heightened sensory perception.

The work of Watanabe et al. opens at least two main lines of research. The first would involve mapping the timescales of brain regions across different neuropsychiatric disorders, including schizophrenia and obsessive-compulsive disorder, to determine where and what type of timescale deviations occur (King and Lord, 2011). This should also be done in healthy individuals to use their timescales as a benchmark. Depending on the location, disturbances ought to have different effects. For example, hub regions play a role in many disorders, and disturbances in their timescales may also evidence their susceptibility to dysfunction (Fornito et al., 2015; Gollo et al., 2018).

The second line of research would explore the possibility of reducing symptoms by manipulating atypical timescales, such as the ones Watanabe et al. observed in people with autism. Although drugs might not be specific enough to selectively act upon precise regions, brain stimulation could be a powerful solution (Figure 1C). For example, superficial cortical regions can be targeted by non-invasive methods such as transcranial magnetic stimulation. Moreover, recent advances suggest that brain stimulation can modify the timescale of the target region, which may be used to modulate intrinsic timescales to mitigate symptoms (Cocchi et al., 2016; Gollo et al., 2017).

Overall, the work of Watanabe, Rees and Masuda reveals how systems-level approaches hold the potential to shift paradigms in psychiatry. Translating these recent results into clinical practice will involve many practical challenges, but they may also be highly beneficial. Although many questions certainly remain, these are crucial advances on the neurobiological basis of autism.
